# Simultaneous LC-ESI-MS/MS Quantification of Levosimendan and Its Metabolites for Therapeutic Drug Monitoring of Cardiac Surgery Patients

**DOI:** 10.3390/pharmaceutics14071454

**Published:** 2022-07-12

**Authors:** Hannah Kipka, Roland Tomasi, Max Hübner, Uwe Liebchen, Christian Hagl, Klaus T. Wanner, Hanna Mannell, Georg Höfner

**Affiliations:** 1 Doctoral Program Clinical Pharmacy, University Hospital, LMU Munich, 81377 Munich, Germany; hanna.mannell@med.uni-muenchen.de; 2 Institute of Cardiovascular Physiology and Pathophysiology, Biomedical Center, LMU Munich, 82152 Planegg, Germany; 3 Department of Anaesthesiology, University Hospital, LMU Munich, 81377 Munich, Germany; roland.tomasi@med.uni-muenchen.de (R.T.); max.huebner@med.uni-muenchen.de (M.H.); uwe.liebchen@med.uni-muenchen.de (U.L.); 4 Walter Brendel Center of Experimental Medicine, LMU Munich, 81377 Munich, Germany; 5 Department of Cardiac Surgery, University Hospital, LMU Munich, 81377 Munich, Germany; christian.hagl@med.uni-muenchen.de; 6 DZHK (German Centre of Cardiovascular Research), Partner Site Munich Heart Alliance, 81377 Munich, Germany; 7 Department of Pharmacy, Center for Drug Research, Ludwig-Maximilians-Universität, 81377 Munich, Germany; klaus.wanner@cup.uni-muenchen.de (K.T.W.); georg.hoefner@cup.uni-muenchen.de (G.H.)

**Keywords:** levosimendan, OR-1896, OR-1855, therapeutic drug monitoring (TDM), LC-ESI-MS/MS, cardiac surgery, metamizole metabolites

## Abstract

Levosimendan is used in severe chronic cardiac insufficiency, also within the peri-operative setting. Real-life pharmacokinetic data in surgical patients is lacking, making therapeutic drug monitoring (TDM) of levosimendan, its pharmacologically active metabolite OR-1896, and its intermediate OR-1855 important. A simultaneous highly sensitive quantification of levosimendan and its metabolites in small-volume samples has not yet been described. Here, levosimendan (LLOQ 0.450 nM), OR-1896, and OR-1855 (LLOQ both 1.0 nM) were successfully quantified by LC-ESI-MS/MS after liquid-liquid extraction in 300 µL of blood. A short C8 column under reversed-phase conditions enabled simultaneous and fast quantification of levosimendan in the negative and the metabolites in the positive ionization mode in a single run within 2 min. Interestingly and unexpectedly, constitutional isomers of levosimendan metabolites with identical mass transitions and similar retention times were observed in surgical patients’ samples, which we identified as the metamizole metabolites 4-aminoantipyrine and 4-acetamidoantipyrine. A longer C8 column and a modified mobile phase enabled selective quantification of all analytes in a single run within 7 min. We developed, validated, and applied highly sensitive LC-ESI-MS/MS methods for simultaneous quantification of levosimendan and its metabolites, enabling efficient TDM of cardiac surgery patients even with additional metamizole administration.

## 1. Introduction

**Figure 1:**
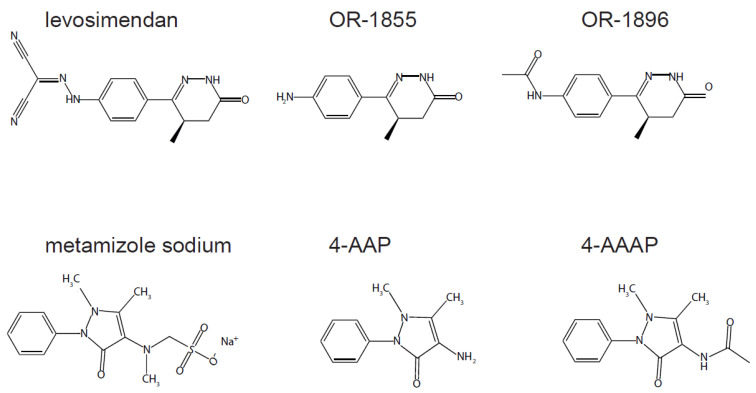
Chemical structures of levosimendan (*M_r_* 280.28; *m/z* 279→227), OR-1855 (*M_r_* 203.24; *m/z* 204→120), OR-1896 (*M_r_* 245.28; *m/z* 246→204), metamizole sodium (*M_r_* 333.34), 4-AAP (*M_r_* 203.24; *m/z* 204→57), and 4-AAAP (*M_r_* 245.28; *m/z* 246→204).

As a calcium sensitizer, levosimendan (Simdax**^®^**) has positive inotropic effects and is therefore used for therapy of severe chronic cardiac insufficiency [1]. In total, 4–7% of the administered levosimendan is metabolized by intestinal bacteria to the metabolite OR-1855, which is acetylated by hepatic N-acetyltransferase (NAT2) to the pharmacologically active OR-1896 (Figure 1) [2]. Whereas levosimendan has a short half-life of one hour, the half-life of the metabolites is 70–80 h. Thus, it is hypothesized that the long-term effects, observed after termination of levosimendan treatment, arise from OR-1855 and OR-1896 [3,4]. Levosimendan is also used in the peri-operative management in cardiac surgery patients, as it is assumed to have a beneficial effect on cardiac function and consequently, postoperative hemodynamics’ [5]. Although numerous phase I–III studies exist, characterizing the pharmacodynamic characteristics and the pharmacokinetic profile of levosimendan as such [6,7,8,9], there is still a lack of real-life data regarding actual levosimendan concentrations within this context. Furthermore, levosimendan is often applied in off-label dosages within this particular setting. Moreover, surgery can additionally affect pharmacodynamics [10]. Whereas high levels of levosimendan can lead to an increased incidence of side effects, such as tachycardia, hypotension, or atrial fibrillation [11] and a higher rate as well as longer duration of vasopressor infusion [12], underdosing may fail to induce a therapeutic effect. Thus, therapeutic drug monitoring (TDM) of levosimendan and its metabolites is essential, enabling adaption of pharmacotherapy according to individual pharmacokinetics. For TDM, easy compound extraction and rapid analysis with high specificity and sensitivity is key. One reason for the limited number of pharmacokinetic studies regarding levosimendan and its metabolites in a real-life setting may be the lack of an efficient method for simultaneous quantification of these compounds. Liquid chromatography coupled with electrospray ionization-based tandem mass spectrometry (LC-ESI-MS/MS) is usually the technique of choice for highly sensitive quantification of multiple analytes with a reasonable throughput in the field of TDM [13]. Although a few protocols for quantification of levosimendan and metabolites are described [14,15,16,17], there is unfortunately none enabling quantification of all three analytes in small-volume blood samples by means of LC-ESI-MS/MS in a single chromatographic run. Hence, we aimed to establish a highly sensitive LC-ESI-MS/MS method for quantification of levosimendan and its metabolites in a single run with a short chromatographic cycle time, which is suitable for TDM in patients undergoing cardiac surgery.

To reach this goal, we used a triple quadrupole mass spectrometer with fast polarity switching capability and ^13^C_6_-labelled analogues of all three analytes as internal standards to achieve a robust quantification of all compounds while taking the different retention behaviours of levosimendan and its metabolites into account.

## 2. Materials and Methods

### 2.1. Materials

Levosimendan was purchased from Sigma-Aldrich (Taufkirchen, Germany). OR-1855, OR-1896, and ^13^C_6_-labelled levosimendan, OR-1855, and OR-1896 were kindly provided by Orion Pharma. Acetonitrile LC-MS grade was purchased from VWR Prolabo (Darmstadt, Germany). 2-Methoxy-2-methylpropane (MTBE), ammonium bicarbonate (NH_4_HCO_3_), and ammonium acetate (CH_3_COONH_4_) were purchased from Sigma-Aldrich (Taufkirchen, Germany). Water was obtained from an ultrapure water system (Sartorius arium pro; Sartorius, Göttingen, Germany). 4-Aminoantipyrine (4-AAP) and 4-acetamidoantipyrine (4-AAAP) were purchased from Merck (Darmstadt, Germany). Due to the different molar masses of levosimendan and its metabolites, concentrations are given in mol/L. All compounds were dissolved in DMSO at a concentration of 10 mM for the stock solution.

### 2.2. Sample Preparation—Liquid-Liquid Extraction

Human blood samples (serum and plasma) were stored at −80 °C. After thawing under room temperature, 25 µL 25 mM CH_3_COONH_4_ (pH 4.75) and 1000 µL MTBE were added to 300 µL sample. The mixture was subsequently vortexed for 30 s, centrifuged (5 min, 1792× *g*), and finally, 900 µL of the organic phase were collected. Extraction was repeated four times, and the collected organic phase evaporated under nitrogen at room temperature, followed by reconstitution of the obtained residue in 300 µL mobile phase. Sample preparation was performed in plastic tubes (Eppendorf, Hamburg, Germany).

### 2.3. LC-ESI-MS/MS

An API 5500 QTRAP triple quadrupole mass spectrometer with a TurboV-ion source (Sciex, Darmstadt, Germany) coupled to an Agilent 1260 HPLC system (Agilent, Waldbronn, Germany), and an SIL-20A/HT autosampler (Shimadzu, Duisburg, Germany) was used. Data acquisition and analysis were performed with Analyst 1.6.3. Levosimendan was quantified in the negative ionization mode at the mass transitions of *m/z* 279.1→227.2, and the metabolites were quantified in the positive ionization mode at the mass transitions of *m/z* 246.3→204.2 (OR-1896), *m/z* 204.2→120.1 (OR-1855), *m/z* 204.0→57.9 (4-AAP), and *m/z* 246.3→204.2 (4-AAAP). The ^13^C_6_ labelled internal standards of levosimendan, OR-1896, and OR-1855 were recorded at *m/z* 285.1→233.2, 252.3→210.2, and 210.2 →126.1, respectively. Quantification was based on the use of corresponding internal standards (for 4-AAP and 4-AAAP, [^13^C_6_] OR-1855 and [^13^C_6_] OR-1896, respectively). For quantification of levosimendan, OR-1855, and OR-1896, a Luna C8 column (30 mm × 2 mm, 3 µm, Phenomenex, Aschaffenburg, Germany) with 5 mM NH_4_HCO_3_ (pH 8.5) and CH_3_CN (76.6/23.4, *v*/*v*) as mobile phase with a flow rate of 350 µL/min at an injection volume of 30 µL was used, employing all internal standards at a concentration of 5 nM. To protect the mass spectrometer for routine analysis, the eluent was diverted to waste from 0 min to 0.2 min and from 1.5 min to 2.0 min. For selective quantification of levosimendan, OR-1855, and OR-1896 in presence of 4-AAP and 4-AAAP, a Zorbax SB-C8 column (150 mm × 4.6 mm, 3.5 µm, Agilent, Waldbronn, Germany) with 5 mM NH_4_HCO_3_ (pH 8.5), CH_3_CN, and CH_3_OH, as mobile phase, (67/23/10, *v*/*v*) with a flow rate of 750 µL/min at an injection volume of 10 µL was used, employing 5 nM [^13^C_6_] levosimendan, 50 nM [^13^C_6_] OR-1855, and 50 nM [^13^C_6_] OR-1896. Both columns were protected with SecurityGuard C8 columns (Phenomenex, Aschaffenburg, Germany) and two inline filters (0.5 µm and 0.2 µm, Idex, Oak Harbor, WA, USA). For source and compound specific parameters, see Supplementary Materials (Table S1). For both methods, the autosampler and column temperature was 20 °C.

### 2.4. Method Validation

Quantification was validated according to the recommendations of the FDA/CDER [18]. The validation procedures for both protocols were performed three times on three different days. If not specified otherwise, serum was used as matrix.

#### 2.4.1. Matrix Effect

Blank matrix (obtained by LLE) as well as solvent was spiked with levosimendan, OR-1855, OR-1896, 4-AAP, and 4-AAAP in quadruplicates at different concentrations. The matrix effect (ME) was characterized by comparison of peak areas in matrix vs. solvent samples and quantitatively assessed according to literature as corresponding percentages of area ratios [18,19].

#### 2.4.2. Linearity

If not mentioned otherwise, preparation of calibrators was performed as described below. Samples from healthy blood donors were spiked before LLE with analytes and internal standards to yield calibration standards. The peak area ratios of analyte and internal standard (y-axis) were plotted against the concentrations of analyte (x-axis). Calibrators investigated at six concentration levels should be within ±15% of nominal concentrations [18]. For linear regression 1/x weighting was used.

#### 2.4.3. Selectivity

For validation six matrix blanks from different individual sources were prepared and investigated [18].

#### 2.4.4. Carryover

After measuring the highest calibrator concentration, a blank sample was measured. Carryover did not exceed 20% of the LLOQ [18].

#### 2.4.5. Accuracy and Precision

If not stated otherwise, samples from healthy blood donors were spiked before LLE with analytes and internal standards to yield quality controls (QCs) samples. QCs were prepared at four concentrations (LLOQ, Low, Mid, High) in quintuplicates. For within-run and between-run analysis, a deviation up to ±15% of nominal concentrations (except at LLOQ ± 20%) was defined as acceptable. For precision, the within-run and between-run analysis was defined as acceptable within ±15% of CV (except LLOQ ± 20%) [18].

#### 2.4.6. Extraction Recovery

Matrix samples spiked with levosimendan, OR-1855, OR-1896, 4-AAP, and 4-AAAP before LLE were compared with those spiked after LLE. Extraction recovery was determined as the area ratio of matrix samples spiked before LLE vs. after LLE [18].

#### 2.4.7. Bench Top Stability

Different concentrations of levosimendan and its metabolites were tested at room temperature and 4 °C. Measurements were performed either directly after sample preparation or 6 h later.

### 2.5. Human Samples

Experiments with serum and plasma were carried out following the rules of the Declaration of Helsinki of 1975, revised in 2013 [20] and was approved by the clinical ethics committee at the medical faculty, LMU (identification code 20-665, approved 11 February 2021; identification code 20-1089, approved 31 August 2020). Human serum and plasma from healthy voluntary blood donors were prepared by centrifugation for 10 min at 1000 *g*. For plasma samples, the tripotassium salt of ethylenediamine tetraacetic acid (“K3EDTA”) was used as an anticoagulant. Anonymized serum samples from patients receiving levosimendan within the peri-operative management at the Department of Cardiac Surgery of the LMU Klinikum were measured retrospectively without preparing replicates due to limited sample volumes. Patients were receiving either 12.5 mg levosimendan or different off-label dosages between 1.25 and 5 mg.

### 2.6. Data Analysis

Log D and p*K*_a_ values were calculated with MarvinSketch 18.1, ChemAxon (Basel, Switzerland). Data are always shown as means ±SD. Data analysis was performed using Excel 2019, Microsoft (Redmond, WA, USA), and Prism 5.0, Graphpad (San Diego, WA, USA).

## 3. Results

### 3.1. LC-MS

**Figure 2:**
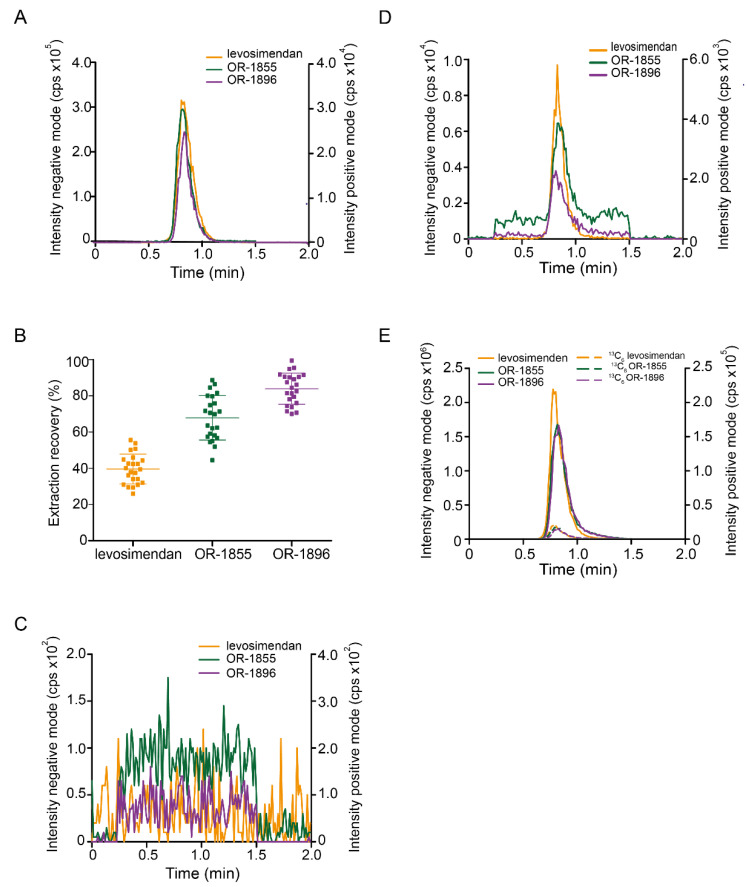
LC-ESI-MS/MS and LLE for levosimendan, OR-1855, and OR-1896. (**A**) MRM chromatogram for levosimendan (5 nM), OR-1855 (10 nM), and OR-1896 (10 nM) in solvent. (**B**) Extraction efficiency from serum samples by LLE for levosimendan, OR-1855, and OR-1896 (n = 23). (**C**) MRM chromatogram of a serum blank. (**D**) MRM chromatogram of levosimendan, OR-1855, and OR-1896 at their LLOQs of 150 pM (levosimendan) and 500 pM (OR-1855 and OR-1896) in serum. (**E**) MRM chromatogram for levosimendan, OR-1855, and OR-1896 at 50 nM (middle QC level) and ^13^C_6_-labelled internal standards (5 nM) in serum. LC-MS for (**A**,**C**–**E**): Luna C8 (30 mm × 2 mm); 5 mM NH_4_HCO_3_ (pH 8.5)/CH_3_CN (76.6:23.4, *v*/*v*); 350 µL/min; MRM: levosimendan (*m*/*z* 279→227), OR-1855 (*m*/*z*, 204→120), and OR-1896 (*m*/*z* 246→204).

Levosimendan, OR-1855, and OR-1896 were recorded at the mass transitions (*m/z*) 279→227 (negative mode), 246→204 (positive mode), and 204→120 (positive mode), respectively, based on previously reported methods [9,16]. For the ^13^C_6_ labelled internal standards, the mass transitions corresponding to the six mass units’ heavier ions (parent as well as fragment ions) in comparison to the non-labelled compounds were found to be the most intensive ones and therefore used for detection. With respect to chromatography, we tried to find isocratic conditions leading to retention factors of at least one for all analytes and sufficient intensities under ESI detection at a short run time. Starting with chromatographic conditions close to those described in previous studies, we could not achieve these aims. Therefore, we investigated various stationary (RP and polar) and mobile phases (varying pH, additives, kind, and portion of organic solvents) and found a short (30 mm) Luna C8 column with 76.6% 5 mM NH_4_HCO_3_ (pH 8.5) and 23.3% CH_3_CN (*v*/*v*), as mobile phase, at a flow rate of 350 µL/min suitable to reach our goal (Figure 2A).

### 3.2. Liquid-Liquid Extraction

Before starting efforts to improve LLE protocols described in literature [2,16], we calculated p*K*_a_ and log D values for levosimendan and its metabolites. Taking this information into account, we hypothesized that a pH of 4.75 in the aqueous phase should be a good compromise for the compounds’ different dissociation behaviours. We obtained, employing MTBE as organic solvent, extraction efficiencies of 39.6% (±8.2%, n = 23), 67.9% (±12.3%, n = 23) and 84.0% (±8.6%, n = 23) for levosimendan, OR-1855, and OR-1896, respectively (Figure 2B).

### 3.3. Method Validation

#### 3.3.1. Matrix Effect

The comparison of spiked serum and solvent samples showed a mean peak area ratio of 114.2% for levosimendan, i.e., an enhancement under matrix conditions. For OR-1855 and OR-1896, peak mean area ratios were 65.7% and 79.1%, respectively, indicating suppression under matrix conditions for both metabolites (Table S2).

#### 3.3.2. Linearity

Blank matrix samples spiked after LLE with analytes and internal standards were used. Linearity was examined in a range from 150 pM to 500 nM for levosimendan and from 500 pM to 1000 nM for the two metabolites, respectively. All calibration functions were characterized by r^2^ values ≥ 0.9995 (Supplementary Materials).

#### 3.3.3. Selectivity

No interfering signals were detectable in matrix blanks (Figure 2C).

#### 3.3.4. Accuracy and Precision

Quality controls (QC) were prepared from blank matrix spiked after LLE with analytes and internal standards. Representative MRM-chromatograms of the analytes at the LLOQ (levosimendan: 150 pM, OR-1855 and OR-1896: 500 pM) as well as for 50 nM are shown in Figure 2D,E. The criteria for accuracy and precision within and between runs were met in all validation series (Table 1).

#### 3.3.5. Bench Top Stability

Extensive studies covering frozen storage, multiple freeze/thaw cycles, autosampler stability, and batch storage before injection regarding levosimendan, OR-1855, and OR-1896 have already been described by Zhang et al. [16], showing that all three analytes are stable under the investigated conditions. Therefore, we additionally only tested the bench top stability at room temperature and 4 °C. The storage for six hours at room temperature or 4 °C did not show any difference to freshly prepared samples (Tables S3–S5).

#### 3.3.6. LLE and LC-ESI-MS/MS with Serum and Plasma Samples

After LC-MS-method validation, we also examined the complete procedure including sample preparation by adding the analytes and their internal standards to 300 µL serum before LLE. LLOQ for levosimendan and the metabolites was 0.45 nM and 1.0 nM, respectively. The calibrators were between 89.1–104.2%, 91.8–107.4%, and 93.2–114.8% of nominal concentrations for levosimendan, OR-1855, and OR-1896, respectively. As plasma samples are also used in daily clinical practice, sample preparation and LC-MS quantification were additionally investigated for plasma samples with the same concentrations for calibrators and quality controls, as described for serum. The results obtained were also consistent with the FDA requirements (Table 1).

### 3.4. Quantification of Levosimendan and Its Metabolites in Patient Serum

**Figure 3:**
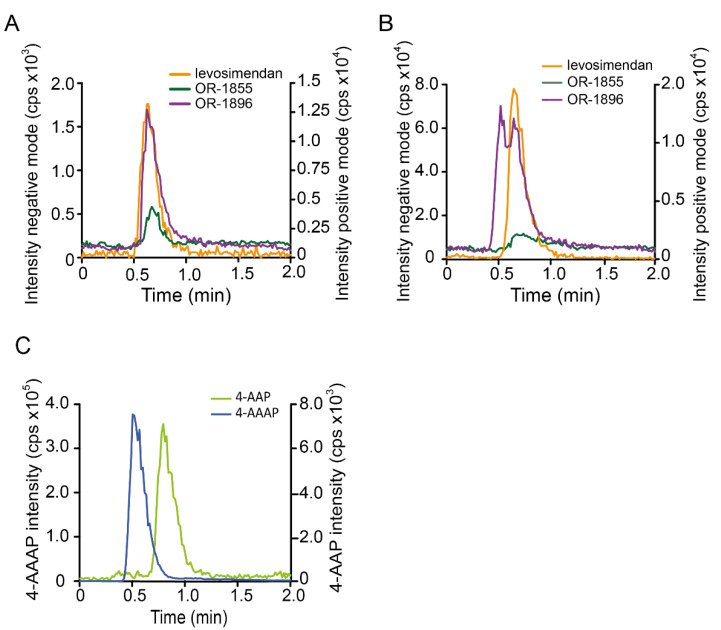
LC-ESI-MS/MS for levosimendan and metabolites in patient samples. (**A**) MRM chromatogram for levosimendan, OR-1855, and OR-1896 of a serum sample taken before surgery from a cardiac patient treated with levosimendan. (**B**) MRM chromatogram of OR-1855 and OR-1896 of a serum sample from a cardiac patient treated with levosimendan with additional metamizole administration taken directly after surgery. (**C**) MRM chromatogram of 4-AAP and 4-AAAP (both 100 nM) in solvent obtained for the mass transitions of OR-1855 and OR-1896, respectively. LC-MS: Luna C8 (30 mm × 2 mm); 5 mM NH_4_HCO_3_ (pH 8.5)/CH_3_CN (76.6:23.4, *v/v*); 350 µL/min. MRM: levosimendan (*m/z*, 279→227), OR-1855 (*m/z*, 204→120), OR-1896 (*m/z*, 246→204), 4-AAP (*m/z*, 204→120), and 4-AAAP (*m/z*, 246→204).

To test if this method was suitable for the detection of levosimendan, OR-1855, and OR-1896 in a clinical sample, serum from a cardiac patient receiving levosimendan, taken before surgery, was first studied. As seen in Figure 3A, all three analytes were detectable at their respective *m*/*z* traces 24 h after levosimendan infusion, whereby the concentration of levosimendan was found to be below the limit defined as LLOQ, i.e., <0.126 ng/mL (450 pM LLOQ). For OR-1855 and for OR-1896, we determined 0.224 ng/mL (1.10 nM) and 2.45 ng/mL (10.0 nM), respectively. To evaluate the suitability of our method for TDM in patients receiving several medications during surgery, serum samples taken directly after surgery from cardiac patients receiving levosimendan were investigated (n = 45; data not shown). The chromatograms in the positive mode, however, showed distinctly distorted peak shapes at the mass transitions (*m*/*z*) 246→204 and 204→120 and slightly shifted retention times. As an example, a chromatogram from one of these samples is shown in Figure 3B, wherein a levosimendan concentration of 0.390 ng/mL (1.39 nM) could be determined. This phenomenon possibly suggests interferences due to an insufficient mass spectrometric selectivity for the levosimendan metabolites. A database search revealed two metamizole metabolites (4-aminoantipyrine, 4-AAP and 4-acetamidoantipyrine, 4-AAAP), which are constitutional isomers of OR-1855 and OR-1896 (Figure 1) [21]. Investigation of 4-AAP and 4-AAAP with our LC-MS settings showed that these compounds are actually detected at the mass transitions (*m*/*z)* 246→204 and 204→120, used to record OR-1855 and OR-1896, at retention times of 0.51 and 0.79 min, respectively (Figure 3C). Indeed, metamizole is routinely used in cardiac surgery patients for pain therapy in our hospital. Investigation of additional mass transitions based on other fragmentations of OR-1855 (*m*/*z* 204→159 and 204→92) and OR-1896 (*m/z* 246→111, 246→120, 246→159, and 246→77) showed distinctly lower intensities (in comparison to *m/z* 246→204 and 204→120) in all cases and revealed only a notable selectivity at *m*/*z* 246→120 for OR-1896 vs. 4-AAAP. Due to the high concentrations of 4-AAAP present in some patient samples, however, an even 50 times higher response for OR-1896 vs. 4-AAAP did not guarantee a sufficiently selective quantification of OR-1896. Therefore, a chromatographic separation of the constitutional isomers was required.

### 3.5. Chromatographic Conditions for Levosimendan Samples Containing Metamizole Metabolites

**Figure 4:**
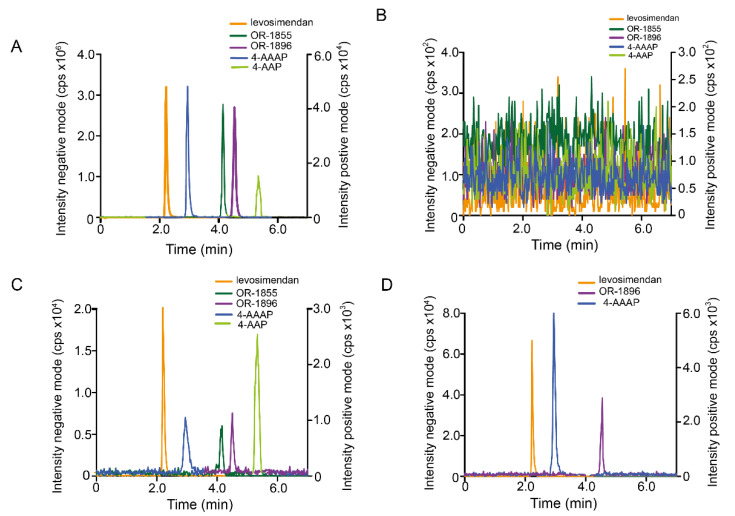
LC-ESI-MS/MS for levosimendan, OR-1855, OR-1896, 4-AAP, and 4-AAAP. (**A**) MRM chromatogram for levosimendan, OR-1855, OR-1896, 4-AAAP (each 75 nM), and 4-AAP (350 nM). (**B**) MRM chromatogram of a serum blank. (**C**) MRM chromatogram for levosimendan, OR-1855, OR-1896, 4-AAP, and 4-AAAP in serum at their respective LLOQs of 450 pM, 1.5 nM, 1.5 nM, 50.0 nM, and 1.35 nM, respectively. (**D**) MRM chromatogram of levosimendan, OR-1896, OR-1855, and 4-AAAP of a serum sample from a cardiac patient (same patient as in 3B) treated with levosimendan and metamizole taken directly after surgery. LC-MS: Zorbax SB-C8 column (150 mm × 4.6 mm); 5 mM NH_4_HCO_3_ (pH 8.5)/CH_3_CN/CH_3_OH (67:23:10, *v/v*); 750 µL/min. MRM: levosimendan (*m/z,* 279→227), OR-1855 (*m/z*, 204→120), OR-1896 (*m/z,* 246→204), 4-AAP (*m/z*, 204→57), and 4-AAAP (*m/z,* 246→204).

Chromatographic separation of the metamizole metabolites 4-AAP and 4-AAAP from OR-1855 and OR-1896 was achieved with 5 mM NH_4_HCO_3_ (pH 8.5), CH_3_CN, and CH_3_OH (67.0/23.0/10, *v/v/v*) as mobile phase employing a Zorbax C8 column (150 mm) within 7.0 min (Figure 4A). This enabled selective quantification by recording of the most intensive mass transitions for OR-1855 (*m/z* 204→120), OR-1896 (*m/z* 246→204), 4-AAP (*m/z* 204→57), and 4-AAAP (*m/z* 246→204).

### 3.6. Liquid-Liquid Extraction for Levosimendan Samples Containing Metamizole Metabolites

Using the established extraction protocol described above, mean extraction efficiencies of 63.9% for 4-AAP and 10.8% for 4-AAAP, respectively, were determined (Table S6).

### 3.7. Method Validation for Levosimendan Samples Containing Metamizole Metabolites

#### 3.7.1. Matrix Effect for 4-AAP and 4-AAAP

The comparison of spiked serum and solvent samples showed mean peak area ratios of 85.0% for 4-AAP and 97.4% for 4-AAAP, indicating a slight suppression under matrix conditions. For levosimendan, OR-1855, and OR-1896, similar matrix effects as found for the first chromatography method were detected (Tables S7 and S8).

#### 3.7.2. Linearity

Linearity was examined for levosimendan from 450 pM to 500 nM, for OR-1855 as well as OR-1896 from 1.5 nM to 500 nM, for 4-AAP from 50 nM to 5000 nM, and for 4-AAAP from 1.35 nM to 5000 nM. Calculated calibration functions were always characterized by r^2^ values ≥ 0.9980 (Supplementary Materials).

#### 3.7.3. Selectivity

No interfering signals were detectable in matrix blanks (Figure 4B).

#### 3.7.4. Accuracy and Precision

QCs including LLOQ (Figure 4C), which was 450 pM for levosimendan, 1.5 nM for OR-1855 and OR-1896, 50 nM for 4-AAP, and 1.35 nM for 4-AAAP, respectively, were investigated. The criteria for accuracy and precision were met for all validation series (Table 2).

### 3.8. Quantification of Levosimendan, OR-1855, OR-1896, 4-AAP, and 4-AAAP in Patient Serum

After validation of the second protocol, its suitability for TDM was confirmed studying samples from surgical patients receiving simultaneous levosimendan and metamizole therapy. As exemplified in Figure 4D for the same sample already shown in Figure 3B under the conditions of the first LC-MS method, the analytes were now clearly separated at their corresponding mass transitions. For the representative sample shown in Figure 4D, serum concentration of levosimendan directly after surgery was 0.40 ng/mL (1.41 nM), for OR-1896 it was 0.88 ng/mL (3.59 nM), and for 4-AAAP it was 1.67 ng/mL (6.81 nM). The concentrations of OR-1855 and 4-AAP were below the LLOQs, i.e., <0.308 ng/mL (1.5 nM) and <10.2 ng/mL (50.0 nM).

## 4. Discussion

TDM of levosimendan is of particular importance in patients with conditions or treatments that can change its bioavailability, such as intensive care or surgical patients, where real-life data regarding its pharmacodynamics and pharmacokinetics is missing at large. In addition, due to frequent off-label dosing in these patients, TDM is an essential tool, which could indeed improve individual adjustments of therapy. To predict the duration of the effect of an applied levosimendan dose, it is necessary to record levels of levosimendan and, additionally, of its pharmacologically active metabolite OR-1896, which is assumed to be responsible for the long-term effect [9]. As generation of OR-1896 is dependent on acetylation of the inactive metabolite OR-1855 and its conversion rate depends on different alleles of NAT2, generating slow or rapid acetylators, respectively [8], measurements of OR-1855 have to be considered relevant as well. Here, we established, validated, and applied two protocols for TDM of levosimendan and its metabolites OR-1855 and OR-1896 in blood samples based on highly sensitive, simultaneous, and fast quantification by LC-ESI-MS/MS in a single chromatographic run employing ^13^C_6_ labelled analogues of all three analytes as internal standards, with the first designed for surgical patients without co-administration of metamizole and the second one for those receiving metamizole. As LC-MS/MS is the favored detection technique for sensitive quantification of small molecule analytes in biological matrices, several groups have tried to make use of it for quantification of levosimendan and its metabolites [2,9,16]. Zhang et al. [16] and Puttonen et al. [9] already reached markedly low LLOQs for levosimendan (0.7 nM), OR-1855 (2.5 nM), and OR-1896 (2.0 nM), but both protocols suffer from some disadvantages impeding implementation for TDM. The LC-ESI-MS/MS approach of Zhang et al. [16] is highly sophisticated and requires two separate chromatographic runs for data acquisition in the negative (levosimendan) and in the positive mode (metabolites). Puttonen et al. published a method based on APCI, able to record all three analytes in a single run, which however consumes high volumes (5–10 mL) of blood samples [9]. As LC-MS detection of levosimendan by APCI was recently found to be less sensitive as compared to ESI by Vlase et al. [17], and furthermore the type of APCI interface employed by Puttonen et al. [9] is no longer available, we focused on an LC-ESI-MS/MS method enabling rapid and highly sensitive quantification of levosimendan and its metabolites in a single chromatographic run, benefiting from a triple quadrupole mass spectrometer allowing fast polarity switching. Realizing this idea, we were able to keep the chromatographic cycle time within 2.0 min and reached even lower LLOQs of 0.45 nM for levosimendan and 1.0 nM for both metabolites in plasma and serum as described so far. Furthermore, we achieved higher extraction efficiencies with LLE compared to the so far most efficient procedure described by Zhang et al. [16] which is in the case of levosimendan with about 40% still only moderate. It has to be considered, however, that this is due to the approach of a single sample preparation step for levosimendan and its metabolites possessing distinctly different acid-base-behavior (levosimendan acidic, metabolites basic) and, therefore, requiring a compromise regarding the extraction conditions. As the intensities observed for equimolar concentrations of our three analytes in LC-ESI-MS chromatograms were sufficiently high for levosimendan but by far lower for the metabolites, a moderate extraction efficiency was more tolerable for levosimendan as compared to its metabolites. Finally, and most importantly in this context, it has to be mentioned that this surely comparably low extraction efficiency does not impair reliable quantification of levosimendan, as low or (even worse) fluctuating extraction efficiencies are in any case fully compensated by the use of ^13^C-labeled levosimendan as internal standard.

Application of this validated method to a sample taken from a cardiac patient receiving levosimendan preoperatively revealed that the established protocol is well-suited for TDM in this field and thus represents an improved tool to monitor levosimendan application. Although the primary intention of the present work was to establish a LC-ESI-MS/MS quantification method for the selected analytes in human serum samples, we additionally used our protocol for human plasma to explore the possible extension of this method to plasma samples. However, a further validation is still needed, before clinical use. At the next step, applying the method on samples collected after cardiac surgery, unexpected interferences at the traces recorded in the positive mode for OR-1855 and OR-1896 were observed. These interferences were shown to originate from two metamizole metabolites, 4-AAP and 4-AAAP, possessing the same molecular formulas as OR-1855 and OR-1896 (i.e., constitutional isomers) as well as fragmentation patterns, leading to product ions with the same *m/z* ratios as the ones to be recorded for detection of the levosimendan metabolites in the MRM mode. Surely, interferences of isobaric compounds can never be excluded in a polypharmaceutical context of surgical patients [22]. However, the situation observed here, where metabolites with identical molecular formulas are generated from different drugs, which in turn fragment—despite distinctly different chemical structures (Figure 1)—to product ions with the same *m/z* ratios as the metabolites of the investigated drug is rather uncommon. Nevertheless, a “mass-selective” quantification of the levosimendan metabolites in the presence of the metamizole metabolites based on different mass transitions was impossible. Therefore, we started a second attempt based on chromatographic separation of OR-1855 from 4-AAP and OR-1896 from 4-AAAP, respectively. After validating this method, we applied it to samples of cardiac surgery patients, and were now able to quantify levosimendan, OR-1855, OR-1896, 4-AAP, and 4-AAAP reliably in a single LC-MS run still in an acceptable chromatographic cycle time of 7.0 min. However, as the measurements were performed with anonymized patient rest samples, there was unfortunately not enough serum to prepare sample replicates, especially since the same samples were used for both protocols. The so far available studies measuring serum or plasma concentrations of levosimendan and its metabolites in cardiac patients do not report an interference with metamizole metabolites or any other applied drugs [23,24]. It can be assumed that this is due to the fact that these studies did not examine surgical patients and that they were performed in countries where metamizole is not approved [25]. Considering the administration of metamizole (dipyrone) as an antipyretic and nonopioid analgesic drug frequently approved for severe pain upon surgery [26] in 19 European as well as in several Latin American and Middle East countries clearly demonstrates the urgent need for a method enabling TDM for levosimendan including surgical patients and those receiving metamizole. Finally, the TDM protocols established here may be used for clinicians and researchers to obtain data on the therapeutic serum or plasma levels of levosimendan and its metabolites, investigating the effect of different doses in children, patients with myocardial dysfunction undergoing cardiac surgery, sepsis, and weaning failure. This in turn may contribute to the development of treatment guidelines and dosing recommendations for the use in clinical practice. Moreover, immediate post-surgery TDM of especially the metabolites OR-1896 and OR-1855, exhibiting long half-lives, in combination with pharmacokinetic modelling may represent a tool to predict therapeutic effects and enable individualized dose adjustments to ensure an optimal therapeutic outcome in patients with reduced ejection fraction undergoing major surgeries.

## 5. Conclusions

We developed, validated, and applied two highly sensitive LC-ESI-MS/MS methods for fast and simultaneous quantification of levosimendan and its metabolites OR-1855 and OR-1896 in low-volume human blood samples. Even in the presence of metamizole metabolites, it is now possible to perform TDM, thus enabling a broad scope of application explicitly including surgical cardiac patients.

## Figures and Tables

**Table 1:** Accuracy (**Acc**, %) and Precision (**Prec,** RSD/%) for QCs of levosimendan, OR-1855, and OR-1896; series 1–3: blank (serum) matrix spiked after LLE; series 4: blank (serum) matrix spiked before LLE; series 5: blank (plasma) matrix spiked before LLE. The validation procedures were performed three times at three different days. QCs were prepared at four concentrations (LLOQ, Low, Mid, High) in quintuplicates.

	Serum															
		LLOQ				Low				Middle				High		
Within Run		Mean	Acc. (%)	Prec. (%)		Mean	Acc. (%)	Prec. (%)		Mean	Acc. (%)	Prec. (%)		Mean	Acc. (%)	Prec. (%)
1. Series	Nom. Conc (nMl)	Calc. Conc (nMl)			Nom. Conc (nM)	Calc. Conc (nM)			Nom. Conc (nM)	Calc. Conc (nM)			Nom. Conc (nM)	Calc. Conc (nM)		
Levosimendan	0.15	0.145	96.7	8.4	0.45	0.449	99.8	7.4	50	52.9	105.8	2.3	500	492	98.3	5.8
OR-1855	0.50	0.471	94.2	9.2	1.50	1.39	92.7	2.6	50	45.2	90.4	7.0	500	467	93.5	4.6
OR-1896	0.50	0.524	104.8	10.1	1.50	1.50	100.0	6.4	50	49.0	98.0	6.6	500	503	100.7	4.4
2. Series																
Levosimendan	0.15	0.143	95.3	12.1	0.45	0.471	104.7	6.3	50	50.6	101.3	5.5	500	533	106.6	5.4
OR-1855	0.50	0.518	103.6	11.3	1.50	1.50	100.0	5.2	50	50.4	100.8	8.6	500	481	96.1	8.3
OR-1896	0.50	0.461	92.2	9.1	1.50	1.61	107.3	4.4	50	52.7	105.4	12.2	500	521	104.3	7.4
3. Series																
Levosimendan	0.15	0.136	90.7	6.3	0.45	0.407	90.4	5.1	50	53.9	107.8	2.1	500	505	101.1	1.8
OR-1855	0.50	0.443	88.6	5.3	1.50	1.51	100.7	7.3	50	53.8	107.6	3.0	500	522	104.4	5.1
OR-1896	0.50	0.522	104.4	6.9	1.50	1.38	92.0	2.9	50	50.8	101.6	4.5	500	477	95.4	5.3
Between run																
Levosimendan	0.15	0.141	94.0	9.1	0.45	0.442	98.2	8.6	50	52.5	105.0	4.2	500	510	102.0	5.6
OR-1855	0.50	0.477	95.4	10.9	1.50	1.47	98.0	6.4	50	49.9	99.6	9.5	500	490	98.0	7.6
OR-1896	0.50	0.502	100.4	10.1	1.50	1.50	100.0	8.0	50	50.8	101.6	8.5	500	501	100.1	6.6
4. Series Including LLE																
Levosimendan	0.45	0.462	102.7	2.8	1.35	1.23	91.1	2.3	50	46.6	93.2	1.8	500	476	95.2	1.8
OR-1855	1.00	0.956	95.6	8.3	4.50	4.39	97.6	3.0	50	50.7	101.4	4.7	500	468	93.6	3.3
OR-1896	1.00	0.889	88.9	3.3	4.50	4.25	94.4	2.0	50	47.1	94.2	1.6	500	457	91.4	1.3
5. Series Including LLE	**Plasma**															
Levosimendan	0.45	0.403	89.6	3.2	1.35	1.55	114.8	0.4	50	56.3	112.6	0.9	500	500	100.0	3.2
OR-1855	1.00	0.990	99.0	11.2	4.50	5.10	113.3	2.4	50	56.3	112.6	2.0	500	550	110.1	4.5
OR-1896	1.00	0.930	93.0	7.5	4.50	4.61	102.4	1.4	50	56.1	112.2	3.5	500	453	90.5	4.1

**Table 2:** Accuracy (**Acc**, %) and Precision (**Prec,** RSD/%) for QCs of levosimendan, OR-1855, and OR-1896, 4-AAP and 4-AAP; series 1–3: blank (serum) matrix spiked before LLE. The validation procedures were performed three times on three different days. QCs were prepared at four concentrations (LLOQ, Low, Mid, High) in quintuplicates.

Serum																
		LLOQ				Low				Middle				High		
Within Run		Mean (nM)	Acc. (%)	Prec. (%)		Mean (nM)	Acc. (%)	Prec. (%)		Mean (nM)	Acc. (%)	Prec. (%)		Mean (nM)	Acc. (%)	Prec. (%)
1. Series	Nom. Conc (nMl)	Calc. Conc (nMl)			Nom. Conc (nMl)	Calc. Conc (nMl)			Nom. Conc (nMl)	Calc. Conc (nMl)			Nom. Conc (nMl)	Calc. Conc (nMl)		
Levosimendan	0.45	0.428	95.1	9.6	1.35	1.39	103.0	2.5	75	84.4	112.5	1.8	300	284	94.7	1.8
OR-1855	1.50	1.52	101.3	6.5	4.50	4.84	107.5	6.3	75	78.8	105.1	6.6	300	316	105.2	5.5
OR-1896	1.50	1.50	100.0	8.0	4.50	4.57	101.6	2.9	75	84.2	112.3	1.4	300	318	106.1	5.1
4-AAP	50	52.3	104.6	5.6	150	162.0	108.0	6.3	350	384	109.8	4.1	750	829	110.5	2.5
4-AAAP	1.35	1.33	98.5	9.0	4.05	3.86	95.3	3.1	75	78.1	104.1	7.4	500	570	114.0	0.7
2. Series																
Levosimendan	0.45	0.438	97.3	1.7	1.35	1.41	104.4	5.0	75	82.3	109.7	3.4	300	301	100.3	2.4
OR-1855	1.50	1.50	100.0	6.6	4.50	4.60	102.2	8.8	75	69.9	93.2	6.3	300	296	98.7	3.4
OR-1896	1.50	1.52	101.3	7.4	4.50	4.36	96.9	3.6	75	70.9	94.5	7.8	300	285	95.1	2.6
4-AAP	50	57.4	114.8	3.0	150	161.0	107.3	5.1	350	391	111.6	5.4	750	745	99.3	9.4
4-AAAP	1.35	1.49	110.4	5.2	4.05	4.53	111.9	1.5	75	86.2	114.9	0.3	500	509	101.7	8.6
3. Series																
Levosimendan	0.45	0.432	96.0	6.5	1.35	1.35	100.0	3.2	75	80.9	107.9	2.4	300	275	91.7	4.4
OR-1855	1.50	1.51	100.7	5.8	4.50	4.45	98.9	4.8	75	74.5	99.3	3.9	300	291	96.9	3.9
OR-1896	1.50	1.39	92.7	10.1	4.50	4.44	98.7	8.2	75	71.9	95.9	2.6	300	283	94.3	4.3
4-AAP	50	55.7	111.4	5.7	150	166.0	110.7	3.5	350	394	112.6	1.5	750	809	107.8	5.5
4-AAAP	1.35	1.48	109.6	4.0	4.05	4.37	107.9	7.0	75	85.0	113.3	1.0	500	570	113.9	0.9
Between run																
Levosimendan	0.45	0.433	96.8	6.3	1.35	1.39	103.0	3.9	75	82.5	110.0	3.0	300	287	95.6	5.0
OR-1855	1.50	1.51	100.7	5.8	4.50	4.63	102.9	7.3	75	74.4	99.2	7.4	300	301	100.3	5.5
OR-1896	1.50	1.47	98.0	8.9	4.50	4.46	99.1	5.4	75	75.7	100.9	9.2	300	296	98.5	6.9
4-AAP	50	55.1	110.2	6.0	150	163.0	108.7	4.9	350	390	111.3	3.9	750	794	105.9	7.4
4-AAAP	1.35	1.43	105.9	7.8	4.05	4.25	104.9	8.2	75	83.1	110.8	5.8	500	549	109.9	6.9

## Data Availability

The data sets generated during the current study are available from the corresponding author on reasonable request.

## Supplementary Materials

The following supporting information can be downloaded at: https://www.mdpi.com/article/10.3390/pharmaceutics14071454/s1; Linearity of LC-MS/MS quantification for levosimendan and its metabolites; Linearity of LC-MS/MS LC-MS/MS quantification for levosimendan and its metabolites in combination with metamizole metabolites; Table S1(a–c): Source and compound-specific parameters for LC-MS; Table S2: Matrix effect of levosimendan, OR-1855, and OR-1896 as described in Section 3.3.1 of the manuscript; Table S3: Bench top stability of levosimendan described in Section 3.3.5 of the manuscript; Table S4: Bench top stability of OR-1855 described in Section 3.3.5 of the manuscript; Table S5: Bench top stability of OR-1896 described in Section 3.3.5 of the manuscript; Table S6: Extraction efficiency of 4-AAP and 4-AAAP as described in Section 3.6 of the manuscript; Table S7: Matrix effect of 4-AAP and 4-AAAP as described in Section 3.7.1 of the manuscript; Table S8: Matrix effect of levosimendan, OR-1855, and OR-1896 as described in Section 3.7.1 of the manuscript.

## References

[1] Lancaster M.K., Cook S.J. (1997). The effects of levosimendan on [Ca^2+^] (i) in guinea-pig isolated ventricular myocytes. Eur. J. Pharmacol..

[2] Antila S., Kivikko M., Lehtonen L., Eha J., Heikkilä A., Pohjanjousi P., Pentikäinen P.J. (2004). Pharmacokinetics of levosimendan and its circulating metabolites in patients with heart failure after an extended continuous infusion of levosimendan. Br. J. Clin. Pharmacol..

[3] Puttonen J., Laine T., Ramela M., Häkkinen S., Zhang W., Pradhan R., Pentikäinen P., Koskinen M. (2007). Pharmacokinetics and excretion balance of OR-1896, a pharmacologically active metabolite of levosimendan, in healthy men. Eur. J. Pharm. Sci..

[4] Erdei N., Papp Z., Pollesello P., Édes I., Bagi Z. (2006). The levosimendan metabolite OR-1896 elicits vasodilation by activating the K ATP and BK Ca channels in rat isolated arterioles. Br. J. Pharmacol..

[5] Shi W.Y., Li S., Collins N., Cottee D.B., Bastian B.C., James A.N., Mejia R. (2015). Peri-operative levosimendan in patients undergoing cardiac surgery: An overview of the evidence. Heart Lung Circ..

[6] Turanlahti M., Boldt T., Palkama T., Antila S., Lehtonen L., Pesonen E. (2004). Pharmacokinetics of levosimendan in pediatric patients evaluated for cardiac surgery. Pediatr. Crit. Care Med..

[7] Põder P., Eha J., Sundberg S., Antila S., Heinpalu M., Loogna I., Planken Ü., Rantanen S., Lehtonen L. (2003). Pharmacokinetic-pharmacodynamic interrelationships of intravenous and oral levosimendan in patients with severe congestive heart failure. Int. J. Clin. Pharmacol. Ther..

[8] Antila S., Pesonen U., Lehtonen L., Tapanainen P., Nikkanen H., Vaahtera K., Scheinin H. (2004). Pharmacokinetics of levosimendan and its active metabolite OR-1896 in rapid and slow acetylators. Eur. J. Pharm. Sci..

[9] Puttonen J., Kantele S., Ruck A., Ramela M., Häkkinen S., Kivikko M., Pentikäinen P.J. (2008). Pharmacokinetics of intravenous levosimendan and its metabolites in subjects with hepatic impairment. J. Clin. Pharmacol..

[10] Kennedy J.M., Van Riji A.M. (1998). Effects of surgery on the pharmacokinetic parameters of drugs. Clin. Pharmacokinet..

[11] Figgitt D.P., Gillies P.S., Goa K.L. (2001). Levosimendan. Drugs.

[12] Gordon A.C., Perkins G.D., Singer M., McAuley D.F., Orme R.M.L., Santhakumaran S., Mason A.J., Cross M., Al-Beidh F., Best-Lane J. (2016). Levosimendan for the prevention of acute organ dysfunction in Sepsis. N. Engl. J. Med..

[13] Shipkova M., Svinarov D. (2016). LC–MS/MS as a tool for TDM Services: Where are we?. Clin. Biochem..

[14] Li S.R., Chen X.Y., Zhang Y.F., Li G.X., Jiang C.M., Zhong D.F. (2008). Determination of levosimendan and its main metabolites in human plasma with HPLC-MS/MS method. Yaoxue Xuebao.

[15] Puttonen J., Kantele S., Kivikko M., Häkkinen S., Harjola V.P., Koskinen P., Pentikäinen P.J. (2007). Effect of severe renal failure and haemodialysis on the pharmacokinetics of levosimendan and its metabolites. Clin. Pharmacokinet..

[16] Zhang J., Gage E.M., Ji Q.C., El-Shourbagy T.A. (2007). A strategy for high-throughput analysis of levosimendan and its metabolites in human plasma samples using sequential negative and positive ionization liquid chromatography/tandem mass spectrometric detection. Rapid Commun. Mass Spectrom..

[17] Vlase L., Kiss B., Bocsan C., Negrutiu S., Vlad I.H., Buzoianu A. (2015). A high-throughput HPLC assay for levosimendan in human plasma with ESI-MS/MS detection. Farmacia.

[18] U.S. Department of Health and Human Services (2018). Food and Drug Administration. Bioanalytical Method Validation Guidance for Industry.

[19] Matuszewski B.K., Constanzer M.L., Chavez-Eng C.M. (2003). Strategies for the assessment of matrix effect in quantitative bioanalytical methods based on HPLC-MS/MS. Anal. Chem..

[20] World Medical Association (2013). World medical association declaration of helsinki, ethical principles for scientific requirements and research protocols. Bull. World Health Organ..

[21] Maurer P.W. (2016). Mass Spectral Library of Drugs Poisons Pesticides Pollutants and Their Metabolites.

[22] Vogeser M., Seger C. (2010). Pitfalls associated with the use of liquid chromatography-tandem mass spectrometry in the clinical laboratory. Clin. Chem..

[23] Kivikko M., Antila S., Eha J., Lehtonen L., Pentikäinen P.J. (2002). Pharmacokinetics of levosimendan and its metabolites during and after a 24-hour continuous infusion in patients with severe heart failure. Int. J. Clin. Pharmacol. Ther..

[24] Jonsson E.N., Antila S., McFadyen L., Lehtonen L., Karlsson M.O. (2003). Population pharmacokinetics of levosimendan in patients with congestive heart failure. Br. J. Clin. Pharmacol..

[25] Andrade S., Bartels D.B., Lange R., Sandford L., Gurwitz J. (2016). Safety of metamizole: A systematic review of the literature. J. Clin. Pharm. Ther..

[26] Cascorbi I. (2021). The Uncertainties of Metamizole Use. Clin. Pharmacol. Ther..

