# Supplementary material for: Simultaneous LC-ESI-MS/MS Quantification of Levosimendan and Its Metabolites for Therapeutic Drug Monitoring of Cardiac Surgery Patients
Source: Pharmaceutics. 2022 Jul 12;14(7):1454. doi: 10.3390/pharmaceutics14071454 (PMC9319272; doi:10.3390/pharmaceutics14071454)
Supplement: Supplementary file 1 [file pharmaceutics-14-01454-s001.zip › pharmaceutics-1786190-supplementary.pdf]

Supplemental Data

Linearity of LC-MS/MS quantification for levosimendan and its metabolites

Supplementary calibration data for Table 1a. The peak area ratios of levosimendan, OR-1855 and OR-1896 vs their internal standards were plotted against the concentrations of levosimendan, OR-1855 and OR-1896, respectively. Three calibration sets in triplicate were performed. Calibration covered a range from 0.15 nM to 500 nM (LLOQ: 0.15 nM, 0.45 nM, 1.5 nM, 5 nM, 50 nM, 250 nM and 500 nM), 0.5 nM to 1 µM (LLOQ: 0.5 nM, 1.0 nM, 4.5 nM, 25 nM, 50 nM, 250 nM, 500 nM and 1000 nM) and 0.5 nM to 1 µM (LLOQ: 0.5 nM, 1 nM, 4.5 nM, 25 nM, 50 nM, 250 nM, 500 nM and 1000 nM) for levosimendan, OR-1855 and OR-1896, respectively. Upon linear regression, the following mean calibration functions, y = 0.2184x + 0.5141, y = 0.1299x + 0.2656 and y = 0.2082x - 0.0624 with r^2 values of 0.9997, 0.9999 and 0.9995 for levosimendan, OR-1855 and OR-1896, respectively, were obtained. The calculated concentrations for all calibrators were between 85.8-115.0% (LLOQ 80.7-101.4%), 86.8-111.2% (LLOQ 80.8-96.6%) and 91.0-114.7% (LLOQ 81.6-101.4%) of nominal concentrations for levosimendan, OR-1855 and OR-1896, respectively. LC: Luna C8 (30 mm x 2 mm); NH_4HCO_3 (5 mM; pH 8.5)/CH_3CN (76.6:23.4, v/v); 350 µL/min. The internal ^{13}C labelled standards were used in a fixed concentration of 5.0 nM in the final samples.

Linearity of LC-MS/MS LC-MS/MS quantification for levosimendan and its metabolites in combination with metamizole metabolites

Supplementary calibration data for Table 2a. The peak area ratios of levosimendan, OR-1855, OR-1896, 4-AAP and 4-AAAP vs their internal standards were plotted against the concentrations of levosimendan, OR-1855, OR-1896, 4-AAP and 4-AAAP, respectively. Three calibration sets in triplicate were performed. Calibration covered a range from 0.45 nM to 500

nM (LLOQ: 0.45 nM, 1.35 nM, 5 nM, 50 nM, 150 nM, 250 nM, 350 nM and 500 nM), 1.5 nM to 500 nM (LLOQ: 1.5 nM, 4.5 nM, 25 nM, 50 nM, 150 nM, 250 nM, 350 nM and 500 nM), 1.5 nM to 500 nM (LLOQ: 1.5 nM, 4.5 nM, 25 nM, 50 nM, 150 nM, 250 nM, 350 nM and 500 nM), 50 nM to 5 µM (LLOQ: 50 nM, 75 nM, 150 nM, 250 nM, 500 nM, 1000 nM and 5000 nM) and 1.35 nM to 5 µM (LLOQ: 1.35 nM, 5.0 nM, 50 nM, 250 nM, 500 nM, 1000 nM and 5000 nM) for levosimendan, OR-1855, OR-1896, 4-AAP and 4-AAAP, respectively. Upon linear regression, the following mean calibration functions, y = 0.2486x + 0.8437, y = 0.01874x – 0.020, y= 0.0202x -0.0188, y = 0.0036x - 0.1429 and y = 0.0711x - 0.420 with r^2 values of 0.9980, 0.9996, 0.9993, 0.9998 and 1.0 for levosimendan, OR-1855, OR-1896, 4-AAP and 4-AAAP respectively, were obtained. The calculated concentrations for all calibrators were in a range from 91.5-111.1% (LLOQ 92.0-97.8%), 94.2-106.2% (LLOQ 92.7-109.3%), and 88.4-107.6% (LLOQ 96.0-105.3%), 85.2-114.2% (LLOQ 90.0-110.2%) and 87.6-114.6% (LLOQ 91.1-111.0%) of nominal concentrations for levosimendan, OR-1855, OR-1896, 4-AAP and 4-AAAP, respectively. LC: Zorbax SB-C8 column (150 mm x 4.6 mm); NH_4HCO_3 (5 mM; pH 8.5)/CH_3CN/CH_3OH (67:23:10, v/v); 750 µL /min. The internal ^{13}C labelled standards were added in a fixed concentration of 5.0 nM for levosimendan and 50.0 nM for OR-1855 and OR-1896.

Table S1a)-c). Source and compound specific parameters for LC-MS

Table 1a) Compound specific parameters

levosimendan	Volt
DP	-120
CE	-28
CXP	-23
OR-1855 & OR-1896	
DP	86
CE	29
CXP	14
4-AAP	
DP	61
CE	53
CXP	8
4-AAAP	
DP	61
CE	19
CXP	18

Table 1b) Source specific parameters for detection of levosimendan, OR-1855 and OR-1896

temperature	650 °C
ion spray voltage	1000 V
curtain gas (N_2)	25 psi
auxillary gas (N_2)	45 psi
nebulizing gas (N_2)	35 psi
collision gas (N_2)	high

Table 1c) Source specific parameters for detection of levosimendan, OR-1855, OR-1896, 4-AAP and 4-AAAP

temperature	650 °C
ion spray voltage	1500 V
curtain gas (N_2)	25 psi
auxillary gas (N_2)	45 psi
nebulizing gas (N_2)	35 psi
collision gas (N_2)	high

Table S2. Matrix effect of levosimendan, OR-1855 and OR-1896 as described in section 3.3.1 of the manuscript

analyte	concentration [nM]	matrix effect [%]
levosimendan	1.0 nM	117.5
		126.9
		112.9
		107.8
mean (±SD)		116.3 (± 8.1)
levosimendan	5.0 nM	115.3
		119.4
		103.8
		96.6
mean (±SD)		108.8 (± 10.5)
levosimendan	10.0 nM	127.7
		134.7
		104.9
		102.1
mean (±SD)		117.4 (± 16.3)
OR-1855	1.0 nM	63.8
		62.8
		77.2
		66.4
mean (±SD)		67.6 (± 6.6)
OR-1855	5.0 nM	59.6
		62.8
		63.0
		63.4
mean (±SD)		62.2 (± 1.8)
OR-1855	10.0 nM	59.8
		68.1
		72.8
		68.8
mean (±SD)		67.4 (± 5.5)
OR-1896	1.0 nM	87.1
		82.9
		84.4
		70.1
mean (±SD)		81.1 (± 7.6)
OR-1896	5.0 nM	87.5
		83.5
		79.4
		75.6
mean (±SD)		81.5 (± 5.1)
OR-1896	10.0 nM	74.0
		69.6
		80.3
		75.4
mean (±SD)		74.8 (± 4.4)

Table S3. Bench top stability of levosimendan described in section 3.3.5 of the manuscript

levosimendan	Area Ratio Analyte vs. int. standard	Area Ratio of t_{0h} vs. Area Ratio t_{6h}
450 pM at RT	1.19E-01	99.2 %
450 pM 6h at RT	1.18E-01	
450 pM on Ice	1.21E-01	93.4%
450 pM 6h on Ice	1.13E-01	
1.35 nM at RT	3.58E-01	92.2%
1.35 nM 6h at RT	3.30E-01	
1.35 nM on Ice	4.78E-01	100.6%
1.35 nM 6h on Ice	4.81E-01	
4.5 nM at RT	1.33E+00	98.5%
4.5 nM 6h at RT	1.31E+00	
4.5 nM on Ice	1.29E+00	96.1%
4.5 nM 6h on Ice	1.24E+00	
50.0 nM at RT	1.47E+01	87.1%
50.0 nM 6h at RT	1.28E+01	
50.0 nM on Ice	1.35E+01	103.7%
50.0 nM 6h on Ice	1.40E+01	
500.0 nM at RT	1.11E+02	91.9%
500.0 nM 6h at RT	1.02E+02	
500.0 nM on Ice	1.03E+02	99.0%
500.0 nM 6h on Ice	1.02E+02	

Table S4. Bench top stability of OR-1855 described in section 3.3.5 of the manuscript

OR-1855	Area Ratio Analyte vs. int. standard	Area Ratio of t_{0h} vs. Area Ratio t_{6h}
1.5 nM at RT	2.67E-01	98.1 %
1.5 nM 6h at RT	2.62E-01	
1.5 nM on Ice	2.70E-01	92.6%
1.5 nM 6h on Ice	2.50E-01	
4.5 nM at RT	7.15E-01	105.2%
4.5 nM 6h at RT	7.52E-01	
4.5 nM on Ice	7.01E-01	99.9%
4.5 nM 6h on Ice	7.00E-01	
13.5 nM at RT	5.30E+00	96.2%
13.5 nM 6h at RT	5.10E+00	
13.5 nM on Ice	2.50E+00	102.8%
13.5 nM 6h on Ice	2.57E+00	
50.0 nM at RT	8.52E+00	96.5%
50.0 nM 6h at RT	8.22E+00	
50.0 nM on Ice	9.41E+00	91.0%
50.0 nM 6h on Ice	8.56E+00	
500.0 nM at RT	7.69E+01	104.8%
500.0 nM 6h at RT	8.06E+01	
500.0 nM on Ice	7.81E+01	99.7%
500.0 nM 6h on Ice	7.79E+01	

Table S5. Bench top stability of OR-1896 described in section 3.3.5 of the manuscript

OR-1896	Area Ratio Analyte vs. int. standard	Area Ratio of t_{0h} vs. Area Ratio t_{6h}
1.5 nM at RT	2.52E-01	100.8%
1.5 nM 6h at RT	2.50E-01	
1.5 nM on Ice	2.24E-01	101.4%
1.5 nM 6h on Ice	2.17E-01	
4.5 nM at RT	7.43E-01	99.5%
4.5 nM 6h at RT	7.47E-01	
4.5 nM on Ice	8.15E-01	97.8%
4.5 nM 6h on Ice	8.33E-01	
13.5 nM at RT	3.46E+00	94.5%
13.5 nM 6h at RT	3.66E+00	
13.5 nM on Ice	1.93E+00	95.5%
13.5 nM 6h on Ice	2.02E+00	
50.0 nM at RT	8.44E+00	100.8%
50.0 nM 6h at RT	8.37E+00	
50.0 nM on Ice	8.09E+00	97.1%
50.0 nM 6h on Ice	8.33E+00	
500.0 nM at RT	7.30E+01	92.5%
500.0 nM 6h at RT	7.89E+01	
500.0 nM on Ice	7.09E+01	101.1%
500.0 nM 6h on Ice	6.92E+01	

Table S6. Extraction efficiency of 4-AAP and 4-AAAP as described in section 3.6 of the manuscript

4-AAP	75 nM	150 nM	350 nM	750 nM
extraction recovery [%]	61.2	69.9	70.1	62.5
	56.9	78.3	54.1	62.5
	61.2	73.2	60.7	55.6
mean [%] (±SD)	59.8 (± 2.5)	73.8 (± 4.2)	61.6 (± 8.0)	60.2 (± 4.0)
4-AAAP	1.35 nM	4.05 nM	75 nM	500 nM
extraction recovery [%]	10.3	13.6	10.2	9.3
	10.1	14.2	12.0	9.2
	8.5	12.6	9.1	10.6
mean [%] (±SD)	9.6 (± 1.0)	13.5 (± 0.8)	10.4 (± 1.5)	9.7 (± 0.8)

Table S7. Matrix effect of 4-AAP and 4-AAAP as described in section 3.7.1 of the manuscript

analyte	concentration [nM]	matrix effect [%]
4-AAP	150.0 nM	79.3
		89.8
		84.1
		81.3
mean ((±SD)		83.6 (± 4.6)
4-AAP	350.0 nM	90.4
		88.0
		89.6
		80.8
mean (±SD)		87.2 (± 4.4)
4-AAP	750.0 nM	85.1
		84.2
		85.6
		81.6
mean (±SD)		84.1 (± 1.8)
4-AAAP	4.05 nM	97.4
		97.0
		102.3
		98.1
mean (±SD)		98.7 (± 2.4)
4-AAAP	75.0 nM	100.2
		98.9
		99.8
		93.6
mean (±SD)		98.1 (± 3.1)
4-AAAP	500.0 nM	97.6
		93.0
		91.1
		99.7
mean (±SD)		95.4 (± 4.0)

Table S8. Matrix effect of levosimendan, OR-1855 and OR-1896 as described in section 3.7.1 of the manuscript

analyte	concentration [nM]	matrix effect [%]
levosimendan	1.35 nM	99.8
		113.2
		98.1
		110.7
mean (±SD)		105.5 (± 7.6)
levosimendan	75.0 nM	107.9
		108.9
		102.3
		105.7
mean (±SD)		106.2 (± 2.9)
levosimendan	300.0 nM	118.7
		117.7
		117.5
		114.3
mean (±SD)		117.1 (± 1.9)
OR-1855	4.5 nM	71.2
		78.8
		74.8
		71.3
mean (±SD)		74.0 (± 3.6)
OR-1855	75.0 nM	68.1
		69.6
		79.1
		65.8
mean (±SD		70.7 (± 5.9)
OR-1855	300.0 nM	72.9
		74.3
		77.6
		82.7
mean (±SD)		76.9 (± 4.4)
OR-1896	4.5 nM	87.3
		92.7
		82.1
		84.6
mean (±SD)		86.7 (± 4.5)
OR-1896	75.0 nM	98.2
		98.4
		94.1
		92.5
mean (±SD)		95.8 (± 3.0)
OR-1896	300.0 nM	83.3
		80.1
		78.0
		82.6
mean (±SD)		81.0 (± 2.4)
